# Microneme Protein 6 Is Involved in Invasion and Egress by *Neospora caninum*

**DOI:** 10.3390/pathogens10020201

**Published:** 2021-02-13

**Authors:** Xianmei Wang, Di Tang, Fei Wang, Gaowei Jin, Lifang Wang, Qun Liu, Jing Liu

**Affiliations:** National Animal Protozoa Laboratory, College of Veterinary Medicine, China Agricultural University, Beijing 100193, China; b20193050458@cau.edu.cn (X.W.); s20153050561@cau.edu.cn (D.T.); s20173050647@cau.edu.cn (F.W.); jingaowei2015@cau.edu.cn (G.J.); wanglifang@cau.edu.cn (L.W.); qunliu@cau.edu.cn (Q.L.)

**Keywords:** *Neospora caninum*, NcMIC6, invasion, egress, virulence

## Abstract

Background: *Neospora caninum*, is the etiological agent of neosporosis, an infection that causes abortions in cattle and nervous system dysfunction in dogs. Invasion and egress are the key steps of the pathogenesis of *N. caninum* infection. Microneme proteins (MICs) play important roles in the recognition, adhesion, and invasion of host cells in other apicomplexan parasites. However, some MICs and their functions in *N. caninum* infection have rarely been reported. Methods: The homologous recombination strategy was used to investigate the function of MIC6 in *N. caninum* infection. Results: ΔNcMIC6 showed a smaller plaque size and weakened capacities of invasion and egress than Nc1. Transcription levels of the egress-related genes CDPK1, PLP1, and AMA1 of ΔNcMIC6 were downregulated. Due to the lack of NcMIC6, virulence of the pathogen in the infected mouse was weakened. The subcellular localization of NcMIC1 and NcMIC4 in ΔNcMIC6, however, did not change. Nevertheless, the transcription levels of MIC1 and MIC4 in ΔNcMIC6 were downregulated, and the expression and secretion of MIC1 and MIC4 in ΔNcMIC6 were reduced compared with that in Nc1. Furthermore, the absence of NcMIC6 weakened the virulence in mice and lower parasite load detected in mice brains. Conclusions: NcMIC6 is involved in host cell invasion and egress in *N. caninum* and may work synergistically with other MICs to regulate the virulence of the pathogen. These data lay a foundation for further research into the function and application of NcMIC6.

## 1. Introduction

*Neospora caninum* is an apicomplexan parasite that causes neuromuscular disease in dogs and reproductive disorders in cattle, impacting the global livestock industry greatly [1]. The ability of apicomplexan parasites to invade and egress from the host cells in a regulated manner is essential for the establishment of infection. As an obligate intracellular parasite, *N. caninum* actively invades the host cells and resides and replicates safely within vacuoles, which are surrounded by the parasitophorous vacuole membrane (PVM) [2]. This apicomplexan parasite shares a unique mode of substrate-dependent motility, which is crucial for invasion and egress. Exocytosis of the micronemes, coupled with the activation of the actomyosin system, is required for egress, which is the part of the parasitic lifecycle involving movement and subsequent invasion of a new host cell [3,4]. Adhesins required for egress are typically termed microneme proteins (MICs) and exist as complexes that are discharged at the apical tip of the parasite [5].

There are a few reports about the microneme proteins of *N. caninum*. NcMIC2 may play a role in the attachment and invasion of host cells by *N. caninum*, and its secretion is regulated by calcium [6]. Considering that anti-NcMIC8 serum effectively inhibits in vitro host cell invasion by tachyzoites, NcMIC8 may be involved in the invasion of host cells by *N. caninum* and may interact and possibly form a complex with NcMIC3 during transportation [7]. Apical membrane antigen 1 (AMA1) is an antigen that cross-reacts with *N. caninum* and *Toxoplasma gondii*, and its antibody inhibits host cell invasion by both these parasites, implying that the process of invasion involving NcAMA1 may be similar to that involving TgAMA1 [8]. *T. gondii* and *N. caninum* tachyzoites exhibit different properties of adhesion to the host cell surface glycosaminoglycans, suggesting that interactions of the host cell surface proteoglycans with *N. caninum* differ from those with *T. gondii*; furthermore, NcMIC3 may also be involved in this process [9,10].

NcMIC1 reportedly binds to the sulfated host cell surface glycosaminoglycans [11] and NcMIC4 exhibits unique lactose binding with chondroitin sulfate A glycosaminoglycans [12]. NcMIC6 was identified in our previous studies [13]. Its secretion depends on the intracellular calcium concentrations, and it interacts with the soluble microneme proteins NcMIC1 and NcMIC4. During invasion, NcMIC6 translocates from the apical tip of a tachyzoite to its posterior region, and it may play roles in parasite motility and host cell invasion. In the present study, NcMIC6 was entirely removed from the parent strain to study its role in *N. caninum* infection.

## 2. Results

### 2.1. Successful Construction of an NcMIC6-Knockout Strain

**Figure 1:**
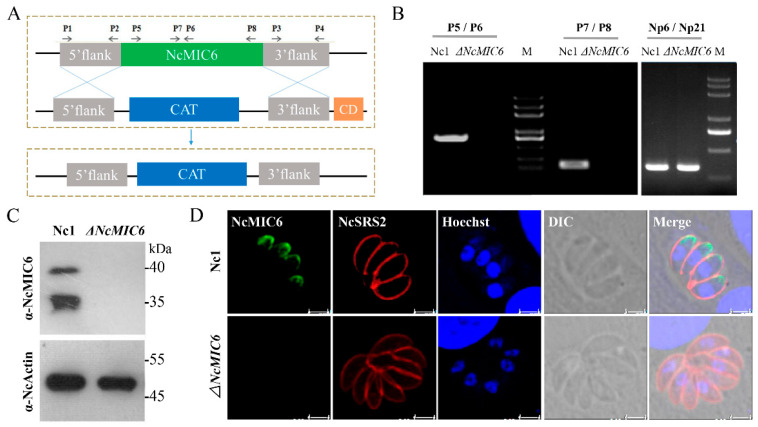
Generation and identification of the ΔNcMIC6 strain. (**A**) Schematic representation of the experimental design of the NcMIC6-knockout strain is shown. A knockout vector (pTCR–CD–MIC6) was constructed to target the complete NcMIC6. CAT, Chloramphenicol acetyltransferase; CD, bacterial cytosine deaminase. (**B**) Genomic PCR identification of the ΔNcMIC6 strain was performed. P5, P6, P7, and P8 were used to amplify different regions of NcMIC6. Np6 and Np21 were used to amplify *N. caninum*-specific Nc5. The position of the primers is shown in the pattern diagram. (**C**) Western blotting was performed on total extracts from ΔNcMIC6 and Nc1 with mouse anti-NcMIC6 antibody. NcActin was used as the control. (**D**) Immunofluorescence assay (IFA) analysis of the expression of NcMIC6 was performed. The parasites were stained with mouse anti-NcMIC6 (green), rabbit anti-NcSRS2 (red), and the nuclear DNA was stained with Hoechst (blue). Each was performed in three independent experiments. Scale-bars, 5 μm.

To understand the role of NcMIC6 in *N. caninum*, we disrupted the endogenous genetic locus using double homologous recombination. We used the complete knockout plasmid pTCR-CD, which included genes of chloramphenicol acetyl transferase (CAT), and the negatively-selected bacterial cytosine deaminase (CD). The plasmid was modified to replace NcMIC6 with the CAT gene (Figure 1A). Following the transfection of the plasmid into Nc1, a stable transgenic line, on chloramphenicol and 5-flucytosine, was selected. The clonal lines were then screened for successful disruption using PCR, meanwhile primers Np6 and Np21 were used to amplify *Neospora caninum*-specific Nc5 gene (Figure 1B). To confirm NcMIC6 deficiency in the transgenic parasites, we analyzed NcMIC6 expression using anti-NcMIC6 antibodies. No expression of NcMIC6 was detected in the knockout strain in Western blotting (Figure 1C) and immunofluorescence assays (IFA) (Figure 1D), indicating the complete deletion of NcMIC6.

### 2.2. NcMIC6 Was Involved in the Invasion by N. caninum

**Figure 2:**
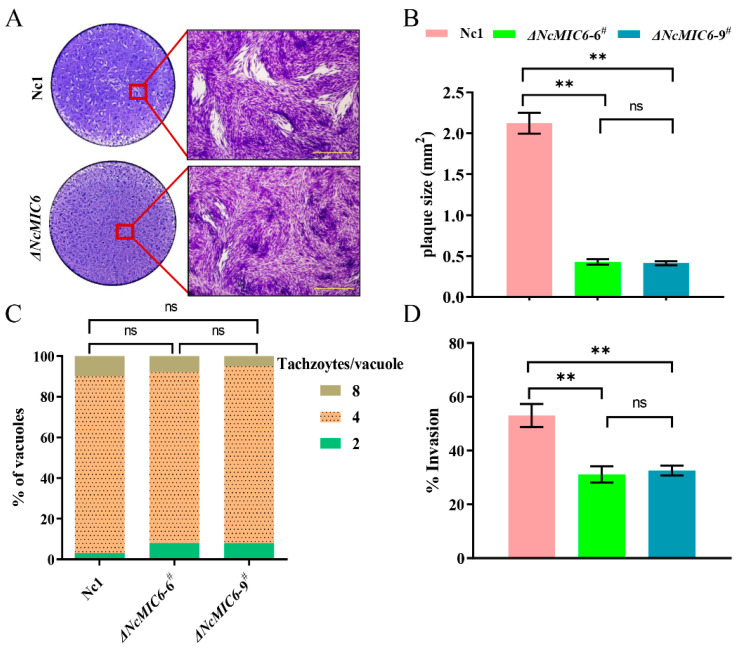
Comparison of the phenotypes of Nc1 and ΔNcMIC6. (**A**) These strains were grown on the Human foreskin fibroblast (HFF) cells for nine days before fixation and staining with crystal violet. Plaque assay showed that ΔNcMIC6 had a smaller plaque size than Nc1; scale-bar, 200 μm. (**B**) Statistics suggested that the plaque area of ΔNcMIC6 (Monoclonal -6^#^ and -9^#^) was significantly smaller than that of Nc1. Data are presented as the mean ± SD from three independent experiments, each performed in triplicate. Plaque size was measured with the Adobe Photoshop CC2018 (Adobe, San Jose, CA, USA) using the Pixel plugin. Data were analyzed with one-way ANOVA with a Tukey test, ns = no significant difference, ** *p* < 0.01. (**C**) Intercellular replication assays showed no significant differences in cell replication of Nc1 and ΔNcMIC6. Data were analyzed with the Chi-square test, ns = no significant difference. (**D**) Invasion assay demonstrated that the ability of cell invasion was obviously reduced after the deletion of NcMIC6. Data are presented as the mean ± SD from three independent experiments, each performed in triplicate. Data were analyzed with One-way ANOVA with Tukey, ns = no significant difference, ** *p* < 0.01.

To investigate the function of NcMIC6, we first analyzed the effect of NcMIC6-knockout on the efficient completion of the asexual lytic lifecycle of the parasite. To monitor NcMIC6′s role overall lytic cycle, we performed a plaque assay by adding 500 freshly purified ΔNcMIC6 and wild-type parasites to fresh Human foreskin fibroblast (HFFs) monolayers. After culturing for nine days, the HFFs were stained using crystal violet. ΔNcMIC6 had a smaller plaque size than Nc1 (Figure 2A,B). In order to detect the proliferation ability of *N. caninum* in the absence of MIC6, we analyzed the number of tachyzoites in each parasitophorous vacuole (PV) (i.e., two, four, or eight tachyzoites) under a fluorescence magnifying lens 24 h after the invasion of host cells. Results of the proliferation assay showed similar replication rates of Nc1 and ΔNcMIC6 (Figure 2C), indicating that NcMIC6 did not affect intracellular parasitic proliferation. The results of the invasion assay clearly demonstrated a decreased ability of the knockout tachyzoites to invade the host cells (Figure 2D). These results suggested that NcMIC6 was involved in invasion during the lytic cycle.

### 2.3. Absence of NcMIC6 Reduced the Expression and Secretion of NcMIC1 and NcMIC4

**Figure 3:**
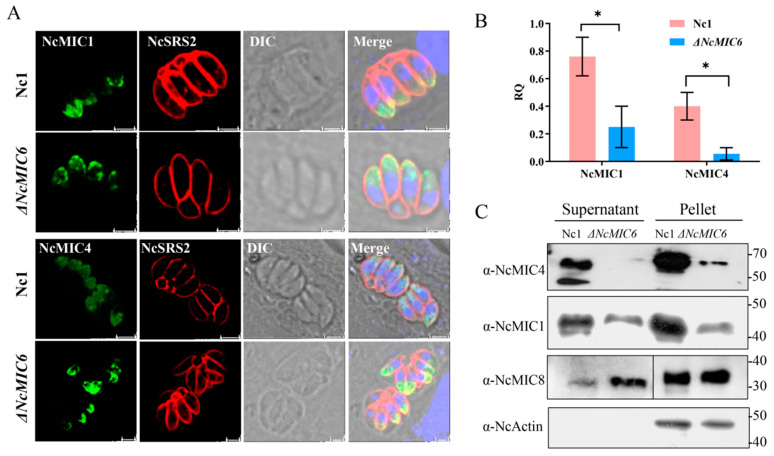
Analysis of NcMIC1 and NcMIC4 in the ΔNcMIC6 strain. (**A**) IFA analysis of the subcellular localization of NcMIC1 and NcMIC4 in ΔNcMIC6 strain was performed. The parasites were stained with mouse anti-NcMIC1, mouse anti-NcMIC4, and rabbit anti-NcSRS2. The results of IFA showed that NcMIC1 and NcMIC4 continued to localize to the micronemes in the ΔNcMIC6 strain, as they did in the Nc1 strain. Scale-bar: 5 μm. (**B**) qPCR revealed that the transcription level of NcMIC1 and NcMIC4 in the ΔNcMIC6 or Nc1 strain. Data were analyzed with one-way ANOVA with a Tukey test, * *p* < 0.05. (**C**) Western blotting of the supernatant and lysates was performed to form the Nc1 and ΔNcMIC6 strains using mouse anti-NcMIC1, mouse anti-NcMIC4, mouse anti-actin, and mouse anti-NcMIC8. A slight decrease in the expression and secretion of NcMIC1 and NcMIC4 protein and a slight increase in NcMIC8 were apparent in ΔNcMIC6. NcActin was used as a loading control. Each was performed in three independent experiments.

NcMIC6 reportedly formed a complex with two other soluble microneme proteins, NcMIC1 and NcMIC4 [13]. To explore whether the deletion of NcMIC6 affected NcMIC1 and NcMIC4, we used separate assays to test their subcellular localization, expression, and secretion. Results of IFA showed that NcMIC1 and NcMIC4 localized to the micronemes in the ΔNcMIC6 strain (Figure 3A). Thus, NcMIC6 was not essential for the correct subcellular localization of NcMIC1 and NcMIC4. qPCR revealed that, compared to Nc1, the transcription levels of NcMIC1 and NcMIC4 in the ΔNcMIC6 strain were down-regulated (Figure 3B). Western blotting was used to evaluate the secretion (in the supernatant) and expression (in the pellet) of NcMIC1 and NcMIC4. Compared with Nc1, ΔNcMIC6 exhibited reduced secretion and expression of NcMIC1 and NcMIC4 (Figure 3C). The increased secretion of NcMIC8 in ΔNcMIC6 may be due to a compensatory mechanism for correct invasion by *N. caninum*.

### 2.4. NcMIC6 Was Engaged in Egress by N. caninum

**Figure 4:**
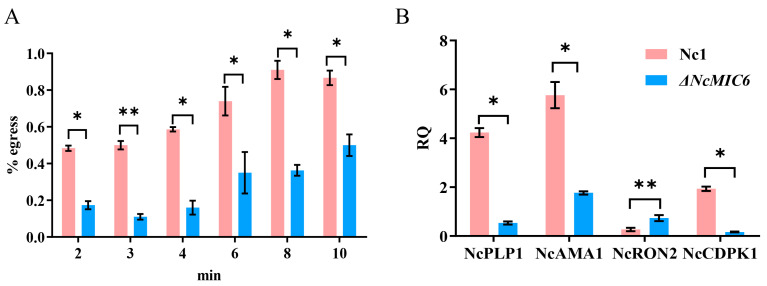
NcMIC6 affects the cellular capability of *N. caninum* to egress. NcMIC6 is required for the calcium ionophore-induced quick egress from the host cells. (**A**) ΔNcMIC6 and wild-type parasite vacuoles were stimulated by the calcium ionophore A23187 for 2–10 min, and the percentage of the egressed vacuoles was determined by IFA. The percentage of the egressed vacuoles markedly decreased in the ΔNcMIC6 strain. (**B**) RT-PCR measured the transcription levels of several crucial molecules related to invasion or egress, including PLP1, AMA1, RON2, and CDPK1. Loss of NcMIC6 significantly downregulated the transcription levels of PLP1, AMA1, and CDPK1. Data are presented as the mean ± SD from three independent experiments, each performed in triplicate. Data were analyzed with one-way ANOVA with a Tukey test, * *p* < 0.05 and ** *p* < 0.01.

Most insights on the egress by coccidian parasites was provided by inducing egress with treatments that directly or indirectly elevated the parasitic cytosolic Ca^2+^ [14]. To determine the efficiency of ionophore-induced egress, we treated ΔNcMIC6 as well as Nc1 with A23187 for different durations. Apparently, ΔNcMIC6, compared to Nc1, showed a strong delay in egress after treatment for different durations (Figure 4A). To identify the pathway that was influenced by the absence of NcMIC6, the transcription levels of several crucial molecules related to egress, including the perforin-like protein 1 (PLP1), apical membrane antigen 1 (AMA1), rhoptry neck 2 protein (RON2), and Ca2+-dependent protein kinase 1 (CDPK1), were measured. Analysis using qRT-PCR revealed that the transcription levels of NcCDPK1, NcPLP1, and NcAMA1 proteins of ΔNcMIC6, compared with those of Nc1, were significantly reduced (Figure 4B). However, NcRON2 was increased (Figure 4B), and this may have been to ensure the correct formation of moving junctions. The generation of NcRON2 by *N. caninum* increased following the decrease in AMA1. Considering that the deletion of MIC6 affected the capability of the parasite cells to egress in vitro, NcMIC6 was involved in egress during the lytic cycle, and it might be associated with different pathways.

### 2.5. The Absence of NcMIC6 Weakened the Virulence in Mice

**Figure 5:**
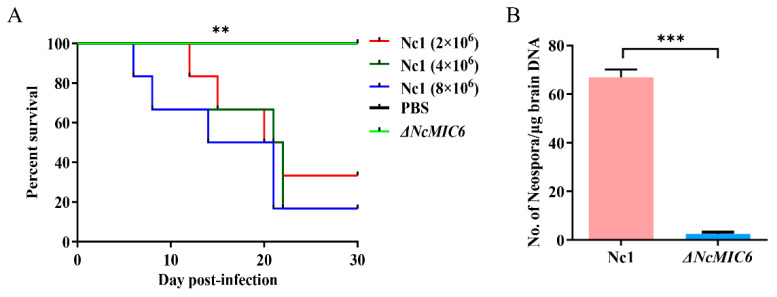
The absence of NcMIC6 causes weakened virulence in mice. (**A**) BALB/c female mice were injected intraperitoneally with 2 × 10^6^, 4 × 10^6^, or 8 × 10^6^ parasites of the two strains (six mice in each group). The groups infected with any dose of ΔNcMIC6 (2 × 10^6^, 4 × 10^6^, or 8 × 10^6^ parasites) displayed a higher survival rate (100%) than those infected with Nc1. A log-rank test was used to analyze significant differences between groups. ** *p* < 0.01. (**B**) BALB/c mice were injected intraperitoneally with 2 × 10^6^ parasites of the two strains (six mice in each group). qRT-PCR was used to determine the cerebral parasite load in the mouse brains. The cerebral parasite load in the mice in the ΔNcMIC6-infected groups was significantly decreased compared with that in the mice in the Nc1-infected groups. Statistical analysis was performed using the life test (life test data = surv) in the statistical analysis system (SAS Institute Inc., USA). The figures are representative of three experiments with similar outcomes. Data were analyzed with one-way ANOVA with a Tukey test, *** *p* < 0.001.

To evaluate the contribution of NcMIC6 to parasite virulence, we measured the survival time of mice infected with ΔNcMIC6 or its parental strain. Mice infected with Nc1 showed different survival rates. The survival rates of the Nc1 low-dose (2 × 10^6^ tachyzoites), middle-dose (4 × 10^6^ tachyzoites), and high-dose (8 × 10^6^ tachyzoites) groups were 33.3%, 16.7%, and 16.7%, respectively. Surprisingly, mice infected with any dose of ΔNcMIC6 (low-dose (2 × 10^6^ tachyzoites), middle-dose (4 × 10^6^ tachyzoites), and high-dose (8 × 10^6^ tachyzoites)) continued to live during the 30-day observation period, displaying a 100% survival rate (Figure 5A). Furthermore, compared with that of the Nc1 group (2 × 10^6^ parasites), the cerebral parasite load of the ΔNcMIC6 group (2 × 10^6^ parasites) was significantly decreased (Figure 5B).

## 3. Discussion

Micronemes are apical specialized organelles that exocytose their contents in a tightly regulated environment, and are critical for egress, gliding, and invasion [15], which seem to be conserved among the apicomplexan parasites. The handling of the dynamic invasion of host cells is preceded by the initial contact of the apical tip with the host cell surface, coinciding with the discharge of micronemes. Exocytosis of the micronemes, which are conveyed over the surface of the parasites, then occurs as part of the interaction between the parasites and host cells [15]. Additionally, microneme proteins are fundamental for the parasites’ gliding motility, which powers movement over biological obstructions and for dynamic entry into the host cell and egress from the infected cells [16]. Several MICs in *N. caninum* have been identified. Previous studies showed that NcMIC6 is a transmembrane microneme protein that localized to the micronemes and translocated from the apical tip of the tachyzoite to its posterior end during invasion into the host cells. This protein possesses three EGF domains, and its secretion is regulated by Ca^2+^, which may be involved in mediating adhesive properties [13]. In our study, complete knockout of MIC6 in *N. caninum* resulted in impairment in the invasion and egress abilities, indicating that NcMIC6 was an important factor in the *N. caninum* lytic cycle, and was involved in the progressive entry of the parasites into the host cells and egress from the infected cells.

Earlier studies also showed that NcMIC6 interacted with NcMIC1 and NcMIC4, forming a complex [13]. In *T. gondii*, the TgMIC1–MIC4–MIC6 complex was the first to be identified and was shown to be critical in invasion [17]. TgMIC1 and TgMIC4 bind to host cells, and MIC6, along with adhesins, establishes a bridge between the host cells and parasite during invasion [18]. The third EGF-like domain (EGF-3) of TgMIC6 plays a role in escorting two dissolvable proteins MIC1 and MIC4 to the micronemes. TgMIC1 and TgMIC4 are mistargeted in ΔTgMIC6 and accumulate in the dense granules and PVs. Unlike those in *T. gondii*, NcMIC1 and NcMIC4 in *N. caninum* continued to localize to the micronemes in the ΔNcMIC6 strain (Figure 4A). Thus, NcMIC6 was not essential for the correct subcellular localization of NcMIC1 and NcMIC4. This suggested a difference in the functional mechanism of invasion by *N. caninum* and *T. gondii*. It was worth noting that we found that the absence of NcMIC6 decreased the expression and secretion of NcMIC1 and NcMIC4, illustrating that MIC6 influenced the complex MIC1-4-6, and was consistent with the emergence and maturation of both MIC1 and MIC4. Decreased MIC1 and MIC4 in the ΔNcMIC6 strain might inhibit binding to the host cells, thereby weakening its invasion capacity.

Insights on parasitic egress in apicomplexans, except Plasmodium and *T. gondii*, have rarely been reported. Dong et al. (2011) found that a premature egression of the sporozoites from the *Eimeria tenella*-infected primary chicken kidney cells or chicken peripheral blood mononuclear cells occurred when the cells were co-cultured with spleen lymphocytes from the *E. tenella*-infected chickens in vitro [19]. Mossaad et al. (2015) showed that calcium ions were involved in the egress of Babesia bovis merozoites from bovine erythrocytes [20]. David, Elliott and Clark (2003) reported that a nonapoptotic death of the host cells was induced on egress of *C. parvum* from the infected cells [21]. 

It is evident that the egress of an intracellular parasite from the host cell is regulated by cyclic nucleotides, phosphatidic acid (PA), and the Ca^2+^ signaling pathway [22]. Individual pathways are tuned to different environmental cues, and multiple pathways may communicate and converge [23]. In the absence of an immune response, parasite growth triggers a breach in the host cell plasma membrane, leading to an efflux of K ionic cues that stimulate parasitic motility. In other circumstances, parasite growth depletes essential host cell resources, and egress requires the secretion of a microneme and an activated actinomyosin machinery [24]. The increase in the intracellular Ca^2+^ by ionophores induces gliding motility and a conoid extrusion [25,26]. Rapidly-dividing *N. caninum* tachyzoites use endodyogeny, a binary replication process, which implicates a single round of DNA replication followed by nuclear mitosis, cytokinesis, and the concomitant assembly and budding of two daughter cells within the mother cell [27,28]. Our data showed that the disruption of NcMIC6 delayed ionophore-induced egress, indicating that the ability of the parasite to egress was weakened. To investigate which pathway was influenced by the absence of NcMIC6, the transcription level of several crucial molecules related to invasion or egress, including PLP1, AMA1, RON2, and CDPK1, were measured. These molecules are conserved in apicomplexans, in which PLP1 ensures lysis of the PVM and host PM [29]. AMA1 forms moving junctions by interacting with the RON2 protein and transducing the force generated by the parasite motor during internalization [30]. CDPK1 is required for the Ca2+-regulated microneme exocytosis and assembly of the actomyosin system; this controls motility, invasion, and egress from the host cells [31,32,33]. In our study, the transcription levels of NcCDPK1, NcPLP1, and NcAMA1 in theΔNcMIC6 strain decreased significantly compared to those in Nc1 (Figure 4B), while NcRON2 was increased (Figure 4B). Considering that AMA–RON pairs reflect the molecular plasticity at the disposal of Apicomplexa to compensate for the dysfunction of the core invasion machinery in *T. gondii* [34], NcRON2 may be increased to compensate for AMA1 dysfunction to ensure the correct formation of the moving junctions. The data showed that NcMIC6 might be associated with different pathways and might eventually affect the capability of the parasite to egress.

Thus, prevention of invasion and egress eventually decreases the virulence of ΔNcMIC6. In mice, decrease in the invasion ability probably prolonged the extracellular time and, hence, it was easier for the host immune system to eliminate the parasites. Meanwhile, the weakened capacity to egress might make it difficult for the parasite to transfer itself among different tissues. What is more, there was much lower parasite load detected in mice brains infected with ΔNcMIC6. These data lay a foundation for further research into the function and application of NcMIC6.

## 4. Materials and Methods

### 4.1. Host Cells and N. caninum Culture

Human foreskin fibroblasts (HFFs) or the African green monkey kidney cells (Vero) were cultured in Dulbecco’s modified Eagle’s medium (DMEM) containing L-glutamine supplemented with 16% or 8% fetal bovine serum (FBS). The Nc1 and NcMIC6-knockout strains were cultured in DMEM supplemented with 2% FBS, penicillin (50 U/mL), and streptomycin (50 μg/mL), and the medium was incubated at 37 °C with 5% CO_2_ in a humidified incubator.

### 4.2. Generation of the NcMIC6-Knockout Strain

To investigate the function of MIC6 in *N. caninum* infection, we used the homologous recombination strategy to generate the NcMIC6-knockout strain. The parental Nc1 strain was used to generate the knockout strain according to a previously described method [35]. The primer sequences used in this study are listed in Supplementary Table S1. Briefly, approximately 1800 bp of the 5ʹ-flanking (P1/P2) and 3ʹ-flanking (P3/P4) sequences of NcMIC6 from the Nc1 genome were amplified. These sequences were flanked with ApaI and XhoI, and EcoRV and SpeI, respectively, and then cloned into the pTCR–CD vector. The knockout plasmid was named pTCR–CD NcMIC6 KO, which included the CAT (chloramphenicol resistance), and CD (bacterial cytosine deaminase, *N. caninum* negative-selected marker gene) genes. We used the plasmid pTCR–CD NcMIC6 KO as a template to PCR amplify linearized pTCR–CAT–NcMIC6 KO, 1 × 10^7^ Nc1 tachyzoites were used to electroporate with 10 µg PCR amplicon. At 24 h after electroporation and recovery, we began drug selection for the chloramphenicol (20 μM). Passages were repeated until the drug-resistant pool became stable (it is usually 5–6 passages until the culture stabilizes). Parasite cloning was screened by limiting dilution and then verified using PCR and Western blotting.

### 4.3. PCR

To screen for the NcMIC6-knockout strain (ΔNcMIC6), we outlined two pairs of primers, based on the NcMIC6 (NCLIV_061760)-coding sequence, for the appropriate amplification of the gene from different clones. P5/P6 and P7/P8 were the preliminary sequences. Nc5 served as an internal reference for the primer match Np6/Np21. The PCR conditions were as follows: 95 °C for 5 min, 30 cycles at 95 °C for 30 s, 56 °C for 30 s, 72 °C for 1 min, and 72 °C for 10 min. The PCR products were then subjected to electrophoresis to visualize the bands.

### 4.4. Western Blotting

Parasites were gathered and purified by filtration through a 5-µm filter, collected by centrifugation at 1400× *g* for 10 min, and washed in phosphate-buffered saline (PBS). Freshly separated parasites were then lysed in the RIPA buffer (50 mM Tris pH 7.4, 150 mM NaCl, 1% Triton X-100, 1% sodium deoxycholate, 0.1% SDS; Beyotime, Shanghai, China) containing the protease inhibitor PMSF (Phenylmethanesulfonyl fluoride, Beyotime, Shanghai, China); next, 7–10 µg of the lysate was utilized for SDS-PAGE (12% *w*/*v*) and then transferred onto polyvinylidene fluoride (PVDF) membranes (Millipore, Burlington, MA, USA). The membranes were blocked with 5% (*w*/*v*) skim milk in PBS. Then, the membranes were incubated at 37 °C for 1 h with the primary antibodies in this study which were mouse anti-NcMIC1, anti-NcMIC4, and anti-NcMIC6 antibody (National Animal Protozoa Laboratory, China Agricultural University, 1:500), rabbit anti-*N. caninum* F-actin subunit beta (Ncactin, National Animal Protozoa Laboratory, China Agricultural University) (1:5000). The secondary antibodies were goat anti-mouse (Sigma, Aldrich, Saint Louis, MO, USA, 1:5000) or anti-rabbit IgG (H + L) horseradish peroxidase (Sigma, Aldrich, Saint Louis, MO, USA, 1:10000). Finally, chemiluminescence reagents (CoWin Biotech Co. Ltd., Beijing, China) were used to visualize reactive bands.

### 4.5. Immunofluorescence Assay

Immunofluorescence assays (IFAs) were performed according to a method described previously [36]. Parasites were seeded onto HFFs that were already arranged on glass coverslips in 12-well plates. Infected cells were incubated at 37 °C with 5% CO_2_ for 24 h, fixed for 30 min in 4% formaldehyde, permeabilized with 0.25% Triton X-100 for 15 min, and then blocked with 3% bovine serum albumin (BSA) for 30 min. Subsequently, the cells were incubated with mouse anti-rNcMIC6 polyclonal antibody [13] stored in our laboratory, diluted to a 1:50 ratio at 37 °C for 1 h and then with, FITC-conjugated goat-anti mouse IgG (H + L) (Sigma, Aldrich, Saint Louis, MO, USA), diluted to a 1:100 ratio with 3% BSA at 37 °C for 1 h. Nuclear DNA was stained with Hoechst33258 (Sigma, Aldrich, Saint Louis, MO, USA) for 5 min. The coverslips were observed, and images were obtained using a Leica confocal microscope system (Leica, TCS SP52, Wetzlar, Germany). Rabbit anti-NcSRS2 polyclonal antibody (preserved in our lab) was used to stain the tachyzoites. The brightness and contrast of the images were adjusted using the LAS AF lite 2.2.0 software, and the images were exported from this software to analyze the NcMIC6 knockout in intracellular parasites.

### 4.6. Plaque Assay

Plaque assay was performed on the HFFs cultured in six-well plates (Corning costar, Cambridge, CA, USA) according to a method described previously [36]. Five hundred freshly collected parasites were seeded onto the HFF monolayers and incubated at 37 °C with 5% CO_2_ for nine days. After nine days, the medium was removed, and the cells were washed three to five times with PBS. The cell monolayers were fixed with 4% formaldehyde for 10 min, stained with 0.2% crystal violet dye for 30 min, washed with deionized water, and visualized by microscopy (Olympus Co., Tokyo, Japan). The six-well plates were scanned under a Canon digital scanner (Model: F917500, Canon, Tokyo, Japan). At least 50 plaques of each strain were chosen arbitrarily, the plaque range was counted utilizing Pixel within the Photoshop C6S program (Adobe, San Jose, CA, USA), and data from three independent tests were compiled.

### 4.7. Proliferation Assay

Freshly separated parasites (1 × 10^6^) were inoculated onto the HFF monolayers in 12-well plates (Corning costar, USA). After 30 min, extracellular parasites were removed by washing three to five times with PBS. After incubation for 24 h, the infected cells were fixed with 4% formaldehyde, and the parasites were stained using rabbit anti-NcSRS2-positive serum following the IFA protocol. The proliferation stages were analyzed by checking the number of tachyzoites in each parasitophorous vacuole (PV) (i.e., 2, 4, or 8 tachyzoites) under a fluorescence magnifying lens; approximately 100 PVs were observed for each strain, as assessed via three independent tests.

### 4.8. Invasion Assay

Freshly isolated parasites (1 × 10^6^) were harvested and seeded onto the HFF monolayers in 12-well plates. After 30 min, the extracellular parasites were removed by washing three to five times with PBS, and the culture plate was then incubated at 37 °C for 24 h with 5% CO_2_. The medium was removed, and the cells were fixed with 4% formaldehyde. To observe parasite invasion and analyze the invasion proportion, IFA was performed following a method described above. Counting was performed under the fluorescence microscope, and data from three independent tests, each performed in triplicate, were compiled. The proportion of the number of infected cells to the total number of cells in one field of view was calculated.

### 4.9. Secretion Assay

The secretion assay was performed using a modified method [37]. Briefly, purified tachyzoites (2 × 10^8^) of Nc1 or ΔNcMIC6 suspended in Hank’s balanced salt solution (HBSS) (100 µL) were transferred to a microfuge tube, 100% ethanol (1 µL) was added, and the mixture was incubated at 37 °C in a water bath for 10 min. After removal of the parasite by centrifugation (1300× *g*, 10 min, 4 °C), the supernatants were processed by SDS-PAGE followed by Western blotting. Meanwhile, NcMIC8 antibody [7] used as a noncomplex microneme protein control and an actin antibody (Ncactin, National Animal Protozoa Laboratory, China Agricultural University) used as the internal control to exclude inadvertent tachyzoite lysis.

### 4.10. Egress Assay

To test the parasites for induced egress, a method similar to that performed for *T. gondii* was performed [38]. Freshly isolated parasites (1 × 10^5^) were harvested and seeded onto the HFF monolayers in 12-well plates. After incubation at 37 °C for 30 min, the extracellular tachyzoites were removed by washing three to five times with PBS, and the medium was then incubated again at 37 °C with 5% CO_2_. After 30 h of growth, the parasites were incubated at room temperature in serum-free DMEM containing A23187 calcium ionophore (5 µM) for 2, 3, 4, 6, 8, or 10 min. The cells were then fixed and stained with the NcSRS2 antibody using IFA, as previously described. A minimum of 100 vacuoles were counted in five randomly chosen fields of view of each well, and the proportion of the egressed versus the non-egressed vacuoles was calculated by counting 100 vacuoles in triplicate measurements from three independent biological replicates.

### 4.11. QRT-PCR

Total RNA was extracted from tachyzoites (2 × 10^7^) of each strain using the TRIzol reagent (Invitrogen, Carlsbad, CA, USA). cDNA was synthesized using the EasyScript First-Strand cDNA Synthesis SuperMix kit (TransGen, Beijing, China). Specific primers were designed for the rhoptry neck protein (NcRON2), the microneme proteins (NcAMA1, NcMIC1, NcMIC4), perforin-like protein 1 (PLP1), Ca^2+^-dependent protein kinase 1 (CDPK1), and the endogenous reference genes (NcActin, Nc18sRNA). The specificity of these primers was evaluated using conventional quantitative real-time PCR (qRT-PCR). qRT-PCR was conducted using the Roche LightCycler System (Biosystems Inc., Foster City, CA, USA) with SYBR Green (Takara Biotechnology, Dalian, China), following the manufacturer’s instructions. The resulting RNA concentrations were normalized using NcActin and Nc18 sRNA, and the relative expression levels of the target genes were analyzed using the ABI Prism 7500 software v2.0.5 (Biosystems Inc., Foster City, CA, USA). The qRT-PCR conditions were as follows: 95 °C for 3 min, 40 cycles at 94 °C for 10 s, 60 °C for 30 s, and 72 °C for 30 s. The relative expression levels of the genes were calculated using the quantification cycle (Cq) value and standardized by the 2-ΔΔCq method. Standard deviation (SD) was calculated from three replicates [39].

### 4.12. Virulence Assay in Mice

Virulence of the parasites was evaluated according to a method described previously [36]. Six-week-old BALB/c female mice were purchased from the Laboratory Animal Center of the Academy of Military. Rodent laboratory chow and tap water were provided, and the mice were maintained under specific pathogen-free conditions and acclimatized for seven days before each experiment. Parasites were injected intraperitoneally into the mice at doses of 2 × 10^6^, 4 × 10^6^, or 8 × 10^6^ tachyzoites (six mice/group). All the infected mice were monitored for clinical signs and mortality every 8 h. The mice were observed daily for 30 days post-infection (dpi). The survival data were compiled from two independent experiments. The mice were humanely euthanized by cervical dislocation, and the cerebral parasite load in the mouse brains was detected at 30 dpi, using qRT-PCR, according to a method described previously [40]. Real-time product formation was monitored using the SYBR Green qPCR Master Mix (Takara, Kyoto, Japan). A 76-bp DNA fragment, corresponding to the *N. caninum*-specific Nc5 was amplified, and the 28S rRNA host gene was quantified to compare the parasite load in different samples.

All animal experiments were approved by the Institutional Animal Care and Use Committee of China Agricultural University (Approval No.: 18049).

### 4.13. Statistical Analysis

Statistical analysis of all the data among groups was performed with GraphPad Prism 8 software (San Diego, CA, USA). Data were analyzed using the two-tailed, one-way ANOVA or Chi-square test or Log-rank test. *p*-values are represented by asterisks. *p* values represented in the figures are as follows: * *p* < 0.05; ** *p* < 0.01; *** *p* < 0.001; ns, not significant. A *p* value < 0.05 was considered statistically significant.

## 5. Conclusions

In summary, functional studies of the microneme protein 6 provided and revealed insights about *N. caninum* invasion and egress from the host cells. This protein may work synergistically with other microneme proteins to regulate the virulence of *N. caninum*. The absence of NcMIC6 weakened the virulence in mice and lower parasite load detected in mice brains. Hence, the parasitic variant ΔNcMIC6 can be potentially used as a preponderant live attenuated vaccine against neosporosis.

## Data Availability

Data supporting the conclusions of this article have been included within the article and its additional files.

## Supplementary Materials

The following are available online at https://www.mdpi.com/2076-0817/10/2/201/s1, Table S1: The sequence of primers.
