# Supplementary material for: Microneme Protein 6 Is Involved in Invasion and Egress by Neospora caninum
Source: Pathogens. 2021 Feb 13;10(2):201. doi: 10.3390/pathogens10020201 (PMC7918358; doi:10.3390/pathogens10020201)
Supplement: Supplementary file 1 [file pathogens-10-00201-s001.pdf]

Table S1. The sequence of primers.

Primer	Sequence
P1	5′ GGGCCCTTGTATCGCTTACGTTGATG 3′
P2	5′ CTCGAGCGAATGCTACCCTCTTGC 3′
P3	5′ GAATTCCATTGTGATCGGTGTTATTG 3′
P4	5′ ACTAGTTCAGTGTTGCTCTGGTGC 3′
P5	5′ GAAGGGTTTCTGTGGCTACAG 3′
P6	5′ AGCAGATCCTTTACTTTCTTC 3′
P7	5′ AAGCCGTGAGGACGAACCCG 3′
P8	5′ AGCGCCCAACAGCAAGAGGC 3′
NP6	5′ CAGTCAACCTACGTCTTCT 3′
NP21	5′ GTGCGTCCAATCCTGTAAC 3′
RON2 F	5′ CGGGACAAGTCAAGTTCTACCA 3′
RON2 R	5′ TTCGCTTCCTGAGCCATC 3′
AMA1 F	5′ TCCACTGACATCGCAACA 3′
AMA1 R	5′ GCCTTAGAATCCGAAACC 3′
PLP1 F	5′ CAGCCGATTGGTGGTTAC 3′
PLP1 R	5′ TTTCGCAAATTCCCTGTAT 3′
CDPK1 F	5′ GAAAGTTGAGAAAGGCAAATAC 3′
CDPK1 R	5′ CGTTGTCAAGGGAGGGTA 3′
Actin F	5′ GACCTTACCGAGTACATGATGAAG 3′
Actin R	5′ CCATCGGGCAATTCATAGGAC 3′
Nc18sRNA F	5′ ATTAGATACAGAACCAACCCAC 3′
Nc18sRNA R	5′ TGAATGATCCGTCGCAGAC 3′
